# The CYP2C19^∗^1/^∗^2 Genotype Does Not Adequately Predict Clopidogrel Response in Healthy Malaysian Volunteers

**DOI:** 10.1155/2013/128795

**Published:** 2013-01-31

**Authors:** Yanti Nasyuhana Sani, Lim Sheau Chin, Lim Luen Hui, Nur Elyana Yazmin Mohd Redhuan Shah Edwin, Goh Teck Hwa, Victor L. Serebruany, Yuen Kah Hay

**Affiliations:** ^1^School of Pharmaceutical Sciences, Universiti Sains Malaysia, 11800 Penang, Malaysia; ^2^Sarawak General Hospital, Clinical Research Centre, 93586 Kuching, Sarawak, Malaysia; ^3^Loh Guan Lye Specialists Centre, Heart Unit, 10400 Penang, Malaysia; ^4^Johns Hopkins University, Johns Hopkins Medicine, Towson, Baltimore, MD 21204, USA

## Abstract

*Background*. The *CYP2C19∗2* allele may be associated with a reduced antiplatelet effect for clopidogrel. Here, we assessed whether *CYP2C19∗2* alleles correlate with clopidogrel responsiveness following the administration of clopidogrel in healthy Malaysian volunteers. *Methods*. Ninety volunteers were genotyped for *CYP2C19∗2* and *CYP2C19∗3* alleles. Forty-five of 90 volunteers were included in the clopidogrel response studies and triaged into three genotypes, namely, *CYP2C19∗1/∗1 *(*n* = 17), *CYP2C19∗1/∗2 *(*n* = 21), and *CYP2C19∗2/∗2 *(*n* = 7). All subjects received 300 mg of clopidogrel, and platelet reactivity was assessed after a four-hour loading utilizing the VerifyNow-P2Y12 assay. Platelet activity was reported using P2Y12 reaction units (PRUs), and nonresponder status was prespecified at PRU ≥ 230. *Results*. Following clopidogrel intake, *CYP2C19∗2/∗2* carriers had a significantly higher mean PRU compared to the *CYP2C19∗1/∗2* and *CYP2C19∗1/∗1* (291.0 ± 62.1 versus 232.5 ± 81.4 versus 147.4 ± 87.2 PRU, *P* < 0.001) carriers. Almost half of the participants (46.7%) were found to be nonresponders (3 were *CYP2C19∗1/∗1*, 11 were *CYP2C19∗1/∗2*, and 7 were *CYP2C19∗2/∗2*). *Conclusion*. In healthy Malaysian volunteers, *CYP2C19∗2* allele was associated with a decrease in platelet responsiveness to clopidogrel. However, clopidogrel nonresponders can be found not only in the carriers of *CYP2C19∗2/∗2*, but also in the carriers of *CYP2C19∗1/∗2* and *CYP2C19∗1/∗1*. The present paper demonstrated that genotype information does not correlate with clopidogrel response, and genotyping may represent a less robust approach compared to platelet activity testing in guiding clopidogrel therapy.

## 1. Introduction

Adequate platelet inhibition plays a key role in the prevention of recurrent ischemic events in patients with acute coronary syndromes (ACSs) undergoing percutaneous coronary intervention (PCI). Accordingly, the use of clopidogrel as a part of the dual-antiplatelet strategy represents a standard of care in clinical practice [1–3]. Recently, the presence of defective alleles of the CYP2C19 enzyme, which is required for the conversion of clopidogrel to its active metabolite, has been associated with lower levels of the active metabolite corresponding in turn to diminished antiplatelet effect and potentially higher rates of adverse cardiovascular events [4, 5]. Consistently, the U. S. Food and Drug Administration (U.S. FDA) has added a black-box warning to the clopidogrel label emphasizing the increased risk of cardiovascular outcomes in patients carrying two loss of function (LoF) CYP2C19 alleles, particularly carriers of CYP2C19 *2 and CYP2C19 *3, which accounts for 85% of reduced function alleles in Whites and 99% in Asians [6]. No similar warning has been given for one LoF CYP2C19 allele carriers. This warning also advocates the availability of genetic testing to identify a CYP2C19 genotype status and the use of genetic testing to aid therapeutic strategy. Following the “black box” warning, two major guidelines in the USA [7] and Europe [8] highlighted the use of genotyping and phenotyping as one part of state-of-the-art modern antiplatelet strategies. To date, the diagnostic roles of genotyping in predicting clopidogrel response and subsequently determining therapeutic strategy in clopidogrel nonresponders are less clear, and the clinical utility in the “real world” is not obvious. Furthermore, most of the studies have been performed on Western subjects with no data available for the Malaysian population, especially the influence of CYP2C19 genotype on clopidogrel response. Therefore, we conducted a study to assess the association between CYP2C19 *2 genotype status and clopidogrel response on healthy Malaysian volunteers. 

## 2. Materials and Methods

### 2.1. Subjects

This study was approved by the University ethics committee (Joint Ethic Committee, School of Pharmaceutical Sciences, Universiti Sains Malaysia Hospital Lam Wah Ee, USM HLWE/IEC/2011/0006) on March 2011, and all of the subjects gave written informed consent to participate in the index study. Briefly, 90 healthy Malaysian volunteers between 21 and 40 years of age were recruited between April and May 2011. Volunteers were comprised of 34 Malay, 49 Chinese, and 7 Indian descendents. The volunteers were recruited from Universiti Sains Malaysia (Penang, Malaysia) and were enrolled based on the following criteria: Malaysian citizenship; age between 21 and 55 years old; normal BMI; and no remarkable medical history. All the volunteers have self reported to be free from any medical conditions, normal blood glucose, creatinine level, and vital signs, including blood pressure and heart rate. Hence, they were considered to be healthy. Of the 90 volunteers genotyped, 45 (18 Malays, 25 Chinese, and 2 Indians) agreed to participate in the clopidogrel response study and were asked to refrain from alcohol, caffeine, and cigarettes 24 hours prior to clopidogrel administration. The subjects were also instructed to stop taking multivitamins, supplements, over-the-counter medications, or any other medications one week prior to drug administration. Study subjects were administered a standard loading dose of 300 mg clopidogrel with 240 mL of plain water. Blood samples were drawn four hours after receiving clopidogrel to test platelet function.

### 2.2. Genotyping

We performed genotyping for the CYP2C19 *2 and CYP2C19 *3 alleles in all of the volunteers. Deoxyribonucleic acid (DNA) was extracted from fresh blood using the *HiYield* Genomic DNA Mini Kit (Real Biotech Corporation, Taipei, Taiwan) and stored at −20°C. All of the subjects were genotyped using polymerase chain reaction (PCR) with *i*-Taq plus DNA polymerase (iNtRON Biotechnology Inc., Kyungki-Do, Korea). Primers used to analyze the CYP2C19 *2 allele included 5′-CAACCAGATCTTGGCATATTG′-3 and 5′-TAAAGTCCCGAGGGTTGTTG′-3. The primers used to analyze the CYP2C19 *3 allele were 5′-CTTCACCCTGTGATCCCACT′-3 and 5′-TGGTTTCTCAGGAAGCAAAAA′-3. The PCR amplification protocol included denaturation at 94°C for 2 min, followed by 40 cycles at 94°C for 30 s, 60°C for 10 s, 72°C for 20 s, and a final elongation at 72°C for 2 min. Negative controls were always included in each PCR analysis. Prior to sequencing, all of the PCR products were purified using LaboPass gel and a PCR Clean-Up Kit (Cosmo Genetech, Seoul, Korea). Direct sequencing was performed with forward and reverse primers using an ABI3730XL capillary-based DNA sequencer (Applied Biosystems, USA) to detect the presence of CYP2C19 *2 and CYP2C19 *3. Sequencing results were interpreted using BioEdit software version 7.0.8.0, which is a biological sequence alignment editor [9]. The CYP2C19 *1 allele was assigned when none of the assayed alleles were present.

### 2.3. Platelet Function

Platelet function was measured using the VerifyNow-P2Y12 test (Accumetrics, San Diego, CA, USA). Results are expressed as P2Y12 reaction units (PRUs), where a lower PRU value is associated with a greater degree of P2Y12 inhibition by clopidogrel and therefore is associated with the expected antiplatelet effect. Nonresponders were prespecified as PRU ≥ 230, and this cutoff point defines a high residual platelet reactivity and has been demonstrated to predict long-term cardiovascular events following percutaneous coronary intervention, including death, myocardial infarction (MI), and stent thrombosis (ST) [10]. 

### 2.4. Endpoints

Endpoints were a PRU value four hours following clopidogrel administration in three genotype groups, CYP2C19 *1/ *1, CYP2C19 *1/ *2, and CYP2C19 *2/ *2, and the total number of nonresponders (PRU ≥ 230) irrespective of their genotype status.

### 2.5. Statistical Analyses

For sample size calculation, we assumed that a 300 mg dose of clopidogrel would result in a PRU absolute difference of 100 PRU with a standard deviation of 50 PRU in P2Y12 ADP-induced platelet reactivity between the CYP2C19 *1/ *1 carriers and CYP2C19 *1/ *2 carriers. This assumption was based on our preliminary unpublished data. It was estimated that at least eight volunteers in each genotype group were required to provide a power of 95% to reach statistically significant differences with a two-tailed alpha of 0.05. The categorical variables are presented as numbers or percentages and were compared using a chi-square test. Continuous variables, presented as means ± SD, were compared using a one-way analysis of variance (one-way ANOVA). After demonstrating a significant difference among the variables with a one-way ANOVA, comparisons among the groups were performed with Tukey's HSD post hoc test to determine which group was significantly different. Statistical significance was *P* < 0.05. All statistical analyses were performed using SPSS for Windows (SPSS 16.0 Family, SPSS Inc., Chicago, IL).

## 3. Results

### 3.1. Baseline Characteristics of the Study Population

Table 1 summarizes the demographic characteristics of the study cohort. Ninety healthy Malaysian volunteers were enrolled in the study (34 Malays, 49 Chinese, and 7 Indians). 37.8% of the volunteers were male, with an average age of 24.1 ± 3.6 years. Mean BMI was 21.3 ± 3.2 kg/m². The vast majority were nonsmokers (98.9%). Of the 90 volunteers genotyped, 45 were included in the clopidogrel response studies (18 Malays, 25 Chinese, and 2 Indians). All 45 subjects were triaged into three genotypes, namely, CYP2C19 *1/ *1 n = 17 (8 Malays, 8 Chinese, and 1 Indian), CYP2C19 *1/ *2 n = 21 (7 Malays and 14 Chinese), and CYP2C19 *2/ *2 n = 7 (3 Malays, 3 Chinese, and 1 Indian).

### 3.2. CYP2C19 Status of the Study Population

**Figure 1:**
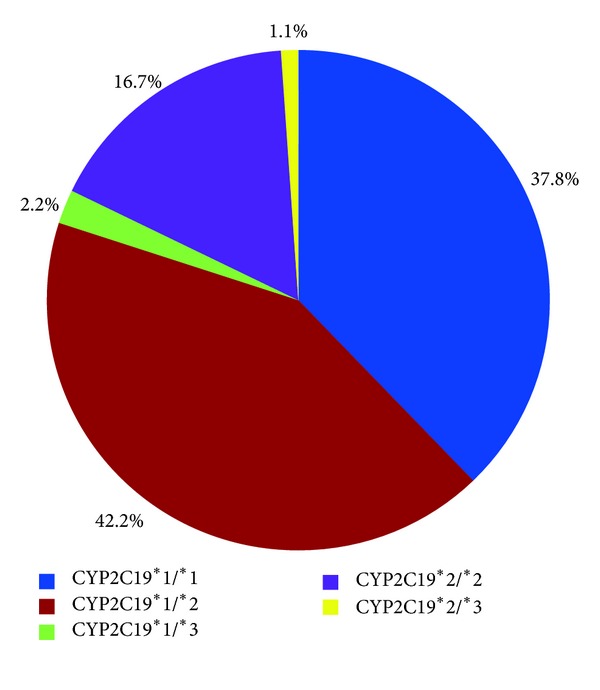
CYP2C19 status of the study population.

Figure 1 shows the prevalence of the CYP2C19 genotype in the 90 volunteers. Of the 90 volunteers genotyped, 37.8% were noncarriers of CYP2C19 *2 or CYP2C19 *3 and therefore were assigned as CYP2C19 *1/ *1 carriers. CYP2C19 *2 was detected in all races (60.0%) and found more in Chinese volunteers (67.3%), followed by Indians (57.1%) and Malays (50.0%). CYP2C19 *3 was detected in only 3.3% of the volunteers. Specifically, 42.2% of the volunteers carried one LoF for the CYP2C19 *2 allele, namely, CYP2C19 *1/ *2. Only 16.7% of the volunteers carried two LoF for the CYP2C19 *2, namely, CYP2C19 *2/ *2. CYP2C19 *1/ *3 was detected in 2.2% of the volunteers, and only 1.1% of the volunteers were detected carrying both defective alleles, namely, CYP2C19 *2/ *3. None of the volunteers carried CYP2C19 *3/ *3.

### 3.3. Impact of CYP2C19∗2 on Platelet Reactivity Following Clopidogrel Administration

**Figure 2:**
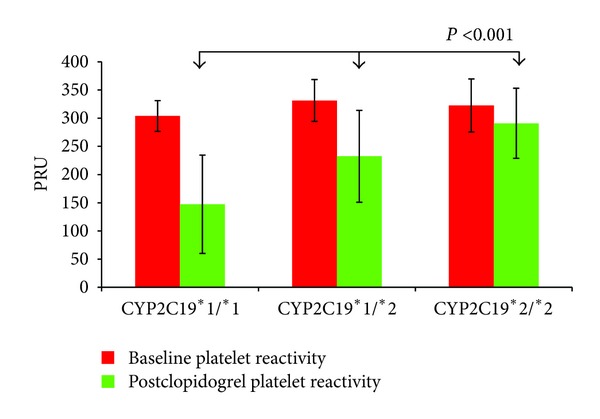
CYP2C19 genotype status and PRU values. Values are expressed as (PRUs) P2Y12 reaction units. The error bars indicate standard deviations of the mean.

Baseline platelet reactivity was essentially similar between the *CYP2C19∗1/∗1*, *CYP2C19∗1/∗2*, and *CYP2C19∗2/∗2* carriers (303.9 ± 27.4 versus 331.4 ± 37.1 versus 322.6 ± 47.0 PRU, respectively, *P* = 0.069). However, platelet reactivity following clopidogrel administration was influenced by the CYP2C19 genotype status with the CYP2C19∗2/∗2 carriers having significantly greater mean platelet reactivity compared to the CYP2C19 *1/ *2 and CYP2C19 *1/ *1 carriers, reflecting an impaired clopidogrel response in the carriers of at least one LoF for the CYP2C19 *2 allele (291.0 ± 62.1 versus 232.5 ± 81.4 versus 147.4 ± 87.2 PRU, respectively, *P* < 0.001) (Figure 2). Post hoc comparisons using Tukey's HSD test indicated that the mean PRU value for the CYP2C19 *1/ *1 carriers was significantly lower from the CYP2C19 *1/ *2 and CYP2C19 *2/ *2 carriers. However, the mean PRU value between the CYP2C19 *1/ *2 and CYP2C19 *2/ *2 carriers did not significantly differ, although we observed a trend towards higher PRU levels in CYP2C19 *2/ *2 carriers compared to CYP2C19 *1/ *2 carriers.

**Figure 3:**
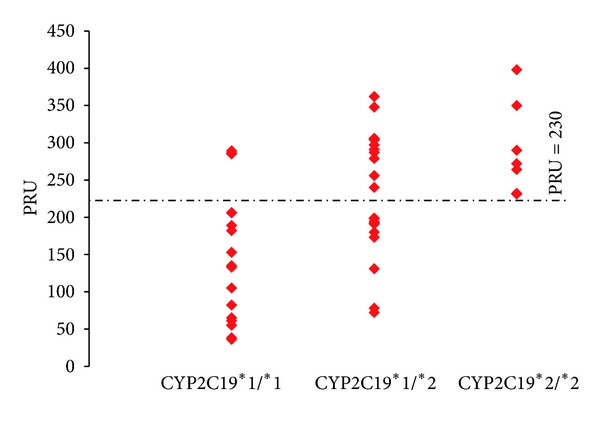
CYP2C19 genotype and PRU distribution. Values are expressed as (PRUs) P2Y12 reaction units. The dotted lines indicate a PRU value equal to 230. The boxes represent an individual PRU value.

For the entire cohort, the proportion of clopidogrel nonresponders (defined by PRU ≥ 230) was 46.7%, irrespective of the CYP2C19 *2 status. Interestingly, three out of the 17 (17.6%) CYP2C19 *1/ *1 carriers were nonresponders, and all 7 (100%) CYP2C19 *2/ *2 carriers were nonresponders. In the case of the CYP2C19 *1/ *2 carriers, 11 out of 21 (52.4%) were also found to be nonresponders. To identify CYP2C19 genotype status of the clopidogrel nonresponders, we constructed a scatter plot consisting of the individual responses in the three strata defined by CYP2C19 genotype status (Figure 3). Interestingly, we found clopidogrel nonresponders not only in the carriers of two LoF for CYP2C19 *2, but also in the carriers of one LoF for CYP2C19 *2 and the noncarriers. All of the CYP2C19 *2/ *2 carriers were nonresponders with the lowest PRU value of 231, whereas three of the CYP2C19 *1/ *1 carriers were identified as nonresponders with a PRU value of 289, 286, and 285. Despite the small sample size for the CYP2C19 *1/ *2 carriers, we observed a broad spectrum of response to clopidogrel, ranging from PRU values as low as 72 to as high as 362, reflecting high variability. A total of 52.4% of the CYP2C19 *1/ *2 carriers were nonresponders and demonstrated overlapped PRU values with the CYP2C19 *2/ *2 carriers, indicating that the diminished clopidogrel antiplatelet effect occurs not only in those having two LoF for CYP2C19 *2 alleles but also in those having one LoF CYP2C19 *2. Most importantly, this observation implies that the CYP2C19 *1/ *2 genotype does not correlate well with phenotype to adequately predict clopidogrel response.

## 4. Discussion

The main findings of the present study can be summarized as follows.

1. The CYP219 *2 allele is consistently present in the Malaysian population, in all main races in Malaysia, namely, Malays, Chinese, and Indians. CYP2C19 *1/ *2 is the most common genotype found in this study population, and a small but significant percentage of the volunteers carried the CYP2C19 *2/ *2 allele. The CYP2C19 *3 allele was also detected in this study population with a lesser degree than the CYP2C19 *2 allele.
2. The CYP2C19 *2 allele was significantly associated with impaired clopidogrel response. The association is not only seen in the carriers of two LoF CYP2C19 *2 carriers, but also in the carriers of one LoF CYP2C19 *2 and CYP2C19 *1/ *1 carriers. This observation implies that a decreased antiplatelet effect of clopidogrel occurs irrespective of CYP2C19 *2 genotype status.
3. A broad spectrum of response was observed in the CYP2C19 *1/ *2 genotype group with half of the carriers clopidogrel nonresponders and another half clopidogrel responders, reflecting that CYP2C19 *1/ *2 genotype status does not correlate well with phenotype to adequately predict clopidogrel responsiveness. Based on this observation, our data suggests that CYP2C19 genotype information alone cannot serve as a reliable surrogate for prediction of platelet reactivity and therefore failed to discriminate between clopidogrel responders or nonresponders, except in those who are carrying two LoF CYP2C19 *2/ *2. Thus, the genotyping approach is probably more predictive in subjects carrying the CYP2C19 *2/ *2 allele compared to the CYP2C19 *1/ *2 allele.

To the best of our knowledge, this is the first paper of an association between CYP2C19 *2 genotype and impaired clopidogrel response performed in a young, healthy Malaysian population. Most importantly, apart from confirming the strong impact of the CYP2C19 *2 allele on the antiplatelet effects of clopidogrel, our paper has also demonstrated that genotyping for CYP2C19 is insufficient to predict clopidogrel response without utilizing the platelet function test.

A timely genome-wide association study has showing that among 400,000 single nucleotide polymorphisms (SNPs), the most significant SNP associated with impaired clopidogrel response was CYP2C19 *2. The carriers of this variant who underwent PCI had reduced protection from clopidogrel in preventing cardiovascular ischemic events or death during one year of followup [11]. A recent meta-analysis which analyzed nine studies predominantly in patients receiving PCI demonstrated that the carriers of at least one LoF for CYP2C19 are at a greater than 50% increased risk for adverse cardiovascular events and a nearly threefold increased risk for ST, whereby the higher risk of ST was demonstrated in the carriers of two LoF for CYP2C19 than the carriers of one LoF [12].

The catastrophic consequences caused by suboptimal ADP-mediated platelet suppression demonstrated in clopidogrel nonresponders were of concern; therefore, more potent P2Y12 inhibitors are needed. Clinical benefits of achieving lower levels of on-treatment platelet reactivity are suggested by two large, randomized controlled trials [13, 14]. In both trials, more potent P2Y12 inhibitors, prasugrel, and ticagrelor were compared with clopidogrel, and both drugs are associated with greater degrees of adenosine diphosphate-mediated platelet inhibition and therefore are associated with a greater suppression of clinical ischemic events. Although the use of more potent P2Y12 inhibitors represents an alternative treatment strategy, this approach is currently unappealing in economic aspects particularly in a country in which affordability and accessibility are still an issue. Based on the aforementioned factor, there is a need to utilize the genetic testing and platelet function tests to determine the best therapeutic strategy. 

More recently, the utility of platelet function testing in guiding antiplatelet therapy was investigated in two prospective trials, namely, (GRAVITAS) with a VerifyNow Assay-Impact on Thrombosis and Safety Gauging Responsiveness [15] and Testing Platelet Reactivity in Patients Undergoing Elective Stent Placement on Clopidogrel to Guide Alternative Therapy with Prasugrel (TRIGGER-PCI) [16]. GRAVITAS suffered from a modest pharmacodynamic effect which resulted in no significant difference of clinical outcomes in patients with high platelet reactivity randomized to standard- or high-maintenance dose of clopidogrel and therefore does not support a treatment strategy for high-dose clopidogrel in patients with high residual activity identified by a single platelet function test in stable patients undergoing PCI. TRIGGER-PCI, which is a trial assessing clinical outcomes in high platelet reactivity patients undergoing elective PCI randomized to prasugrel or clopidogrel, on the other hand suffered from low rates of events and was recently halted. More recently, a pharmacogenetic approach was introduced in the (RAPID-GENE) using an individualized strategy based on genetic evaluation reassessment of antiplatelet therapy trial [17]. This proof-of-concept pharmacogenetic trial has demonstrated that none of the carriers of at least one LoF CYP2C19 *2 or the noncarriers in the rapid genotyping arm had PRU > 234 compared with the standard therapy arm, reflecting the utility of a pharmacogenetic approach to tailor therapy based on genetic information. However, there are no large-scale clinical studies to date demonstrating that adjustment of antiplatelet therapy based on a pharmacogenetic approach improves clinical outcomes.

Pharmacogenetic issues of clopidogrel are significant in Malaysia as the CYP219 *2 allele is highly prevalent in the Malaysian population. It was reported that the prevalence of the carriers of two LoF CYP2C19 *2 and/or CYP2C19 *3 was 5.6% in Malaysian Malays, 19.1% in Malaysian Chinese, and 10.0% in Malaysian Indians [18]. CYP2C19 *2 was reported as the major CYP2C19 variant found in the Malaysian population with the CYP2C19 *1/ *2 genotype mostly detected, especially in Chinese. CYP2C19 *3 was reported in a smaller percentage than CYP2C19 *2 but not an insignificant number. Our study reported a similar trend, although the sample size was smaller than the previously published data. Multiple lines of evidence show that the presence of CYP2C19 *2 is associated with impaired antiplatelet effects of clopidogrel with the association not only seen in healthy volunteers [19, 20] but also in patients [21, 22]. Most of the studies were performed in Caucasians, and only a handful of studies involved the Asian population. Our findings of CYP2C19 variants and clopidogrel response are largely consistent with studies of others and contribute additional data for the Asian population and unarguably the first report involving the Malaysian population. Interestingly, we observed few clopidogrel nonresponders not only detected in the carriers of at least one LoF CYP2C19 *2, but also in the noncarriers. This finding reflects the fact that CYP2C19 *2 only account for a certain percentage of variability in clopidogrel response. A possible explanation for this finding might be that other nongenetic or genetic factors could play additional roles. It was reported that the CYP2C19 *2 genotype only accounts for approximately 12% of the variation in clopidogrel response in patients cohort, whereas another 10% of the variability is contributed by clinical risk factors with the majority of these causes remaining unexplained [11]. All CYP2C19 *2/ *2 carriers were nonresponders reflecting diminished clopidogrel responsiveness in the carriers of two LoF CYP2C19 *2, which represents a group of poor metabolizers. It was reported in at least two prospective studies that even with the maintenance clopidogrel doses up to 300 mg [23] and loading doses up to 900 mg [24], CYP2C19 *2/ *2 carriers responded poorly, suggesting a profile of complete resistance to clopidogrel. We also observed a broad spectrum of response within the CYP2C19 *1/ *2 genotype group. As such, more than half of the CYP2C19 *1/ *2 carriers were clopidogrel nonresponders, and the remaining carriers were responsive to clopidogrel. Similarly, it was reported in a study using stable coronary artery disease patients that approximately 18–25% of the CYP2C19 *1/ *1 carriers and 38–43% of the CYP2C19 *1/ *2 carriers demonstrated high platelet reactivity following clopidogrel administration [25]. 

There are two major implications for these findings. The U.S. FDA emphasized increased risk of adverse cardiovascular outcomes in patients with two LoF CYP2C19 variants, and no similar warning was given for those with one LoF CYP2C19. Our study clearly demonstrated that significant portions of CYP2C19 *1/ *2 carriers have overlapping PRU values with CYP2C19 *2/ *2 carriers and therefore are regarded as nonresponders. In light of this observation, we think that the decreased clopidogrel response does not exclusively belong to the carriers of two LoF CYP2C19 *2 only, but also in one LoF CYP2C19 *2 carriers, therefore carrying a similar increased risk of adverse cardiovascular events. Secondly, the wide response variability observed in CYP2C19 *1/ *2 carriers implies that genotype information may fail to identify a large portion of patients with high platelet reactivity or patients with optimal platelet reactivity and therefore unable to discriminate between clopidogrel responders and nonresponders. This is the main reason why genotype information is not a robust surrogate or marker for predicting pharmacodynamic response to clopidogrel. However, our data suggest that for persons carrying two LoF CYP2C19 *2/ *2, genotype correlates well with phenotype to adequately predict clopidogrel nonresponders. Therefore, if one were to personalize clopidogrel therapy on the basis of genotype information alone, many CYP2C19 *1/ *2 carriers who are potentially responding to clopidogrel are unnecessarily switched to the new generation of P2Y12 inhibitors. The role of genotyping may be important for CYP2C19 *2/ *2 carriers for selecting intensified clopidogrel therapy or more potent P2Y12 inhibitors. As a large proportion of CYP2C19 *1/ *2 carriers were reported in the Malaysian population, and if drug costs are taken into account, especially when generic clopidogrel will substantially reduce the cost, it seems that the roles of phenotyping outperform genotyping. Furthermore, phenotyping provides significant advantages as it measures the final drug response irrespective of genetic or nongenetic factors which contribute to clopidogrel unresponsiveness.

## 5. Limitation of Study

This study has several limitations that require mentioning. This study is a single-center study involving only a small number of healthy volunteers. Nonetheless, the results showed that a high proportion of the Malaysian population might be resistant to clopidogrel therapy. We did not measure the plasma clopidogrel active thiol metabolite and therefore were not able to correlate pharmacodynamic response with pharmacokinetic data. Our results are merely laboratory findings without associated clinical outcomes, since we did not assess the clinical impact of the reduced clopidogrel response in patients with altered CYP2C19 activity. 

## 6. Conclusions

In healthy Malaysian volunteers, CYP2C19 *2 is associated with a marked decrease in platelet responsiveness to clopidogrel, but genotype information alone cannot serve as a reliable surrogate or marker for platelet reactivity especially in the carriers of one LoF CYP2C19 *2. Therefore, genotyping is a less robust approach in predicting clopidogrel response compared to platelet studies. These data warrant further investigation in a larger sample size and in the clinical setting of a randomized trial.

## Figures and Tables

**Table 1:** Demographic characteristics of the study population.

Characteristics	All volunteers (*n* = 90)	Malays (*n* = 34)	Chinese (*n* = 49)	Indians (*n* = 7)	*P* value
Age (yrs)	24.1 ± 3.6	22.4 ± 1.4	25.3 ± 4.4	24.3 ± 1.8	0.055
Male sex	34 (37.8)	16 (47.1)	15 (30.6)	3 (42.9)	0.254
BMI (kg/m²)	21.3 ± 3.2	22.6 ± 3.4	20.1 ± 2.4	21.4 ± 4.2	0.014
Smokers	1 (1.1)	1 (2.9)	0 (0)	0 (0)	0.424
CYP2C19*2	54 (60.0)	17 (50.0)	33 (67.3)	4 (57.1)	0.348
CYP2C19*3	3 (3.3)	2 (5.9)	1 (2.0)	0 (0)	0.877

Values are mean ± SD or *n* (%).
